# Unraveling the impact of microwave-assisted techniques in the fabrication of yttrium-doped TiO_2_ photocatalyst

**DOI:** 10.1038/s41598-023-51078-0

**Published:** 2024-01-02

**Authors:** Adam Kubiak, Michał Cegłowski

**Affiliations:** Faculty of Chemistry, Adam Mickiewicz University, Poznan, Uniwersytetu Poznanskiego 8, 61614 Poznan, Poland

**Keywords:** Materials science, Nanoscale materials, Chemical synthesis

## Abstract

In this study, we investigate the role of microwave technology in the fabrication of yttrium-doped TiO_2_ through a comparative analysis of hydrothermal techniques. Microwave-assisted hydrothermal synthesis offers advantages, but a comprehensive comparison between microwave-assisted and conventional methods is lacking. Therefore, in our investigation, we systematically evaluate and compare the morphological, structural, and optical properties of yttrium-doped TiO_2_ samples synthesized using both techniques. The X-ray diffraction (XRD) patterns confirm the anatase tetragonal structure of the synthesized TiO_2_-Y systems, while the larger ion radius of yttrium (Y^3+^) compared to titanium (Ti^4+^) presents challenges for yttrium to incorporate into the TiO_2_ lattice. The X-ray Photoelectron Spectroscopy (XPS) revealed a significant difference in the atomic content of yttrium between the TiO_2_-Y systems synthesized using microwave-assisted and conventional methods. This finding suggests that the rapid microwave method is more effective in successfully doping TiO_2_ with rare earth metals such as yttrium. The photo-oxidation of carbamazepine (CBZ) using TiO_2_-Y systems demonstrated high efficiency under UV-LED light. Microwave-synthesized TiO_2_-Y demonstrates improved photo-oxidation efficiency of CBZ, attributed to enhanced absorption, charge transfer, surface area, and crystallite size. Overall, the microwave-synthesized TiO_2_-Y systems showed promising performance for the photo-oxidation of CBZ, with improved efficiency compared to conventional synthesis methods.

## Introduction

Titanium dioxide has garnered significant attention in research due to its remarkable photocatalytic properties, chemical stability, and wide availability^1^. However, it also has inherent limitations that impede its full potential. One such drawback is its wide band gap, which restricts its photocatalytic activity to the ultraviolet (UV) region^2,3^. Additionally, TiO_2_ exhibits a high recombination rate of photogenerated electron–hole pairs, diminishing its overall photocatalytic efficiency. These limitations hinder the effective utilization of photogenerated charge carriers for desired photocatalytic reactions^4^. To overcome these challenges, researchers have turned to dope TiO_2_ nanostructures with rare earth metals. Rare earth metals offer the potential to enhance the surface, optical, and photocatalytic properties of TiO_2_. They possess vacant 4f. orbitals that readily interact with functional groups, and their incorporation introduces more oxygen defects, effectively suppressing electron–hole recombination during transfer^5,6^. Moreover, rare earth doping increases impurity levels, leading to more efficient utilization of photo-induced carriers. Doping with rare earth ions, particularly those with 4f. electron configurations, significantly enhances adsorption capacity and the rate of photogenerated carrier separation^7,8^. Among the rare earth elements, yttrium (Y) is a representative candidate. The crystal structure of TiO_2_ favors the replacement of Ti^4+^ ions with Y^3+^ ions over substituting O_2_^−^ with any other anion^9,10^. This preference arises from differences in charge states and ionic radii. Yttrium possesses unique electronic and chemical characteristics that can influence the structural, optical, and photocatalytic properties of TiO_2_^11,12^. Researchers have explored different synthesis methods to achieve precise control over the doping process, including conventional hydrothermal techniques and microwave-assisted hydrothermal synthesis.

Hydrothermal synthesis is a method that utilizes high temperatures and pressures in an aqueous environment to facilitate the formation of desired materials^13,14^. Khan et al.^15^, emphasized the benefits of this method by illustrating its ability to synthesize Sm^3+^ doped rutile TiO_2_ nanorods decorated with GdFeO_3_ nanorods for efficient CO_2_ conversion and organic pollutants degradation. Additionally, this technique is versatile, as seen in its production of mediator-free hollow BiFeO_3_ spheres and porous g-C_3_N_4_ photocatalysts for both CO_2_ conversion and Alizarin Red S degradation. These examples undoubtedly highlight its significant potential^16^. However, one challenge in hydrothermal synthesis is maintaining uniform temperature distribution throughout the reaction mixture^17^. Temperature gradients can occur within the reactor, potentially leading to variations in material properties or incomplete reactions. To address this issue, microwave-assisted hydrothermal synthesis has emerged as a promising approach, particularly in the fabrication of TiO_2_-based materials^18–20^. Microwave technology provides rapid and efficient heating, resulting in accelerated reaction kinetics and reduced synthesis times compared to conventional heating methods. The selective heating achieved through microwave irradiation can improve homogeneity and enhance material properties^21,22^. Nevertheless, it is important to consider the potential drawbacks associated with microwave-assisted hydrothermal synthesis. The rapid and intense heating provided by microwaves can sometimes induce unintended side reactions or undesired product selectivity^23,24^. Localized overheating or thermal gradients caused by microwave radiation may affect reaction pathways and product distribution. Moreover, it should be noted that microwave synthesis relies on the selective heating of materials by microwave radiation^25^. Achieving efficient heat transfer throughout the sample can be challenging, especially for larger volumes or materials with low microwave absorption^26^. Non-uniform heating can lead to uneven reactions and incomplete product formation. Therefore, careful optimization of microwave parameters and reaction conditions is necessary to mitigate these challenges and maximize the benefits of microwave-assisted hydrothermal synthesis. Understanding the trade-offs and potential limitations is essential in effectively utilizing this technique to synthesize yttrium-doped TiO_2_ and other materials^27–29^.

This publication presents a novel study that investigates the role of microwave technology in the fabrication of yttrium-doped TiO_2_. It does so by conducting a head-to-head comparison with conventional hydrothermal techniques. Through a comprehensive analysis, the study examines the impact of microwave-assisted hydrothermal synthesis on the structural characteristics, optical properties, and photocatalytic performance of yttrium-doped TiO_2_ materials. By comparing the outcomes with those obtained through conventional hydrothermal methods, the research offers valuable insights into the impact of microwave technology on the synthesis process and resulting material properties. The findings contribute to our understanding of how microwave technology can enhance the fabrication of yttrium-doped TiO_2_ materials. This study represents an important advancement in the field of TiO_2_ photocatalysis, highlighting the scientific and technological progress achieved through the integration of microwave technology in the fabrication of yttrium-doped TiO_2_.

## Materials and methods

### Materials

Titanium(IV) chloride (97%), urea (p.a.), yttrium(III) chloride hexahydrate (99%), ammonium oxalate (99%), silver nitrate (> 99%), tert-butyl alcohol (99%) benzoquinone (p.a,), and carbamazepine (analytical standard) were purchased from Merck. All reagents were of analytical grade and used without any further purification. The water used in all experiments was deionized.

### Synthesis of TiO_2_-Y systems by hydrothermal treatments

The TiO_2_-Y combinations were created using both traditional and microwave techniques. Initially, a 1 wt.% solution of titanium(IV) chloride was prepared in distilled water with an ice-water bath. Then, 100 cm^3^ of the TiCl_4_ solution was moved to an IKA reactor (Ika Werke GmBH, Germany), and 1 g of urea was added with continuous stirring for 15 min. The resulting solution was then shifted to a hydrothermal reactor (Parr Instrument Co., USA) or a microwave reactor (CEM Discover SPD 80, USA) for heat treatment. In the hydrothermal approach, the heat treatment settings were adjusted to T = 200 °C and t = 12 h, whereas in the microwave treatment, the settings were T = 200 °C, t = 1 min, and P = 300W. After the process, the reactor was allowed to cool to room temperature, and the resulting substance was washed thrice with deionized water before being dried at 60 °C for 6 h.

To generate the TiO_2_-Y systems, the procedure started by dissolving 50 mg of YCl_3_·6H_2_O (for 1 wt.% yttrium) and 100 mg of urea in 100 cm^3^ of water, which was then placed in the IKA reactor (Ika Werke GmBH, Germany). Simultaneously, a suspension of pre-synthesized TiO_2_ in water (1 g of TiO_2_ in 100 cm^3^) was prepared. The TiO_2_ suspension was combined with the yttrium precursor solution and stirred for 30 min to ensure uniformity. The resulting mixture underwent hydrothermal microwave treatment, with the following heat treatment conditions applied: T = 200 °C, t = 12 h for a conventional hydrothermal process; T = 200 °C, t = 5 min, P = 300 W for microwave treatment. After the process was completed, the reactor was allowed to cool to room temperature, and the resulting material was washed three times with deionized water. Subsequently, it was dried at 60 °C for 6 h. Each set of synthesized samples was labeled using the formula mentioned below:

1 $$Method \;abbreviatio{n}_{X}\%Y,$$

where *Method abbreviation*—M in case the microwave processing; H—for a conventional hydrothermal treatment, *X%*—the amount of yttrium (wt.% Y = 0.25, 0.5, 1, 1.5, and 2).

### Characterization of TiO_2_-Y systems

The physicochemical characterization of the fabricated TiO_2_-Y systems included the crystalline structure (X-ray diffraction—XRD), BET measurement (low-temperature N_2_ sorption), X-ray photoelectron spectroscopy (XPS), morphology (transmission electron microscopy—TEM), surface composition (energy dispersive X-ray analysis—EDX) and optical properties (diffuse reflectance spectroscopy—DRS and photoluminescence—PL). The detailed data on the conducted physicochemical analyses are presented in Supplementary Materials.

### Photocatalytic activity

#### Light sources

This study utilized a UV light emission diode (LED) light source that made use of LED strips emitting light at a wavelength of 395 nm. Specifically, a 5050 SMD low-voltage LED strip from MEiSSA, Poland, featuring 60 LEDs per meter and generating 7.2 W per meter, was employed in the construction of the reactor. To achieve a power output of 10 W, a 1.5-m length of the same LED strip was used. The LED strip was positioned within an aluminum radiator, accompanied by a fan connected to an IoT thermoregulator, ensuring consistent experimental conditions. The entire setup was linked to a ballast manufactured by Mean-Well, Taiwan, with the power output being verified to align with the intended value.

#### Photo-oxidation test

To begin the experiment, a solution of carbamazepine was prepared by dissolving 20 mg of CBZ in 1 dm^3^ of water. For each experimental iteration, 100 cm^3^ of the prepared CBZ solution was utilized. This solution was then mixed with 100 mg of photocatalyst in a photoreactor. To minimize the impact of ambient light, the entire process was conducted within a dark enclosure. The resulting mixture was thoroughly stirred without exposure to light for 30 min to establish adsorption/desorption equilibrium. Subsequently, the UV-LED lamp was switched on, and the reaction mixture was exposed to irradiation. At regular intervals of 30 min (up to a total of 240 min, after which irradiation was stopped), a 3 cm^3^ portion of the suspension was collected and filtered using a syringe filter manufactured by Macherey–Nagel in Germany. The filtered solution was then analyzed using a UV–Vis spectrophotometer (Shimadzu UV 2020, Japan) across the wavelength range of 200–700 nm, with the spectrum of demineralized water serving as the reference baseline. The maximum absorbance of the pollutant was observed at a wavelength of 265 nm. To determine the photocatalytic activity of the samples, the calibration curve method was employed, using the equation y = 0.0341x + 0.0139, where x represented the CBZ concentration and y denoted the maximum absorbance value.

#### Kinetic study

The kinetics of CBZ photooxidation were assessed using a pseudo-first-order kinetic model. This model postulates that the degradation rate is directly proportional to the surface coverage (θ) of CBZ, expressed as follows:

2 $$r=\frac{dC}{dt}=k\theta =\frac{kK{C}_{0}}{1+K{C}_{0}+{K}_{s}{C}_{s}}$$

Here, *k* represents the reaction rate constant, 'θ' denotes the surface coverage by CBZ, *K* and *K*_*s*_ are the adsorption coefficients for CBZ and water, respectively, C_0_ stands for the initial concentration of CBZ, and C_s_ represents the concentration of water. The concentration of water, C_s_ remains nearly constant and is significantly higher than the concentration of CBZ. Consequently, we can express Eq. (3) in the following form:

3 $$ln\frac{{C}_{t}}{{C}_{0}}=-{k}_{1}t$$

In Eq. (3), *k*_1_ signifies the first-order rate constant, and *t* is the time of irradiation.

#### Verification of the degradation mechanism using scavengers

The study aimed to comprehend the role of charge carriers and reactive oxygen species in the photocatalytic reaction, providing insights into the mechanism of degradation of organic contaminants in the presence of the synthesized TiO_2_-Y systems. To evaluate the photocatalytic activity, the method described in section “Photo-oxidation test” was employed, with the introduction of scavenger solutions in appropriate quantities. The concentrations of the scavenger solutions were adjusted to achieve a level of 20 mg/dm^3^ for CBZ. Specifically, ammonium oxalate was selected as the scavenger for holes (h^+^), AgNO_3_ for electrons (e^−^), tert-butyl alcohol for free hydroxyl radicals (^*^OH), and benzoquinone for superoxide radical anions (^*^O_2_^−^).

#### MS measurement

To better identify the byproducts resulting from the photocatalytic oxidation of cbzusing variously prepared TiO_2_-Y samples, we conducted MS measurements. The ESI–MS spectra were acquired using a Bruker amaZon SL ion trap instrument (Germany), which was equipped with an electrospray ion source operating in infusion mode. The sample solution was introduced into the ionization source at a flow rate of 5 μL min^−1^ via a syringe pump. The instrument operated in the *enhanced resolution mode* within a mass range of 50–30,000 m*/z*, scanning at a rate of 8100 m*/z* per second. The capillary voltage was set to + 4.5 kV, with an endplate offset of −500 V. The ion source temperature was maintained at 80 °C, while the desolvation temperature was set to 250 °C. Nitrogen served as the cone gas, and helium as the desolvating gas, with flow rates of 800 L h^−1^ and 50 L h^−1^, respectively. The mass spectrometer was operated in both positive and negative ionization modes.

## Results and discussion

### Characterization of yttrium-doped TiO_2_ systems

**Figure 1:**
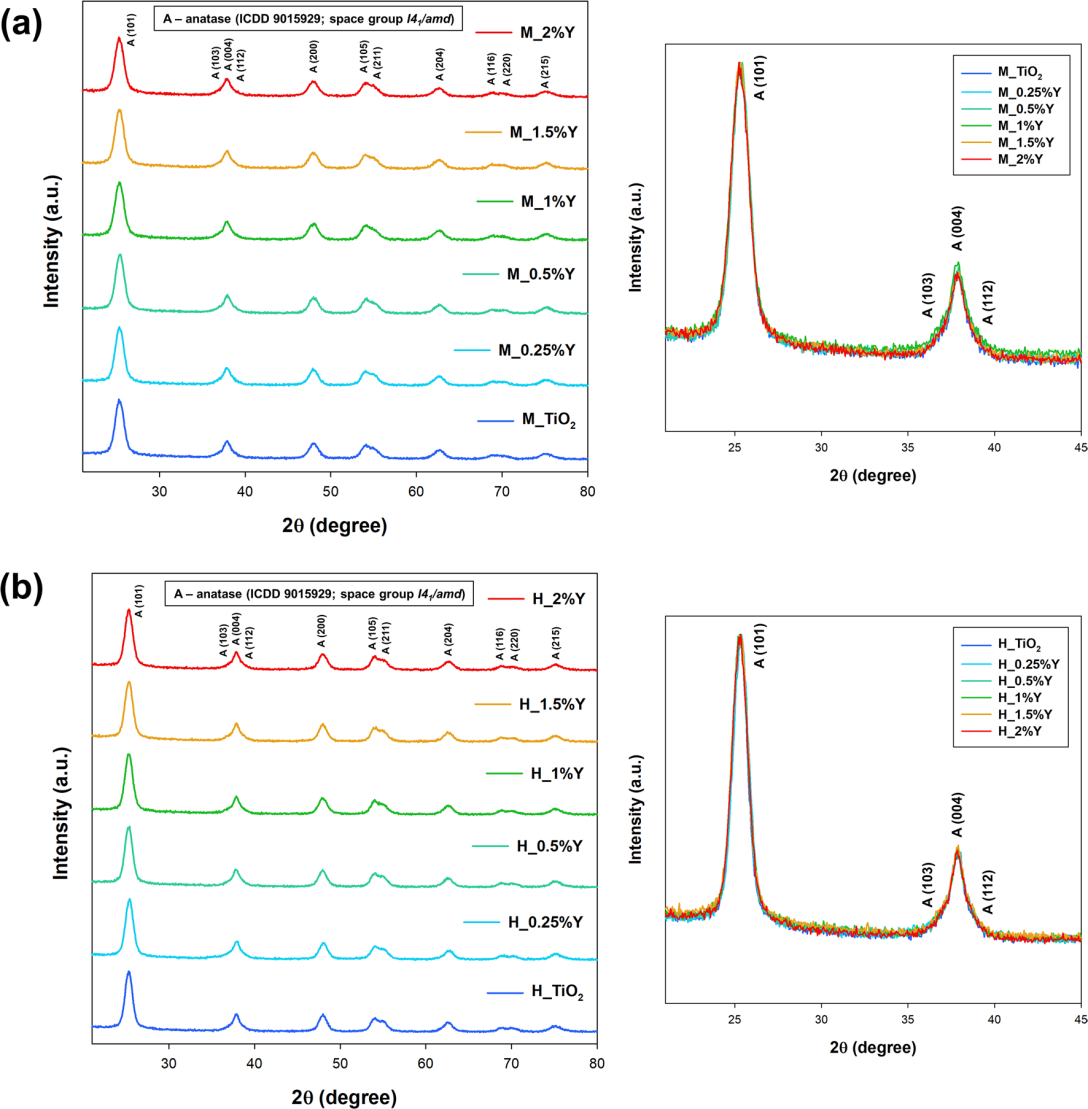
The XRD patterns for TiO_2_-Y systems synthesized by (**a**) microwave and (**b**) conventional treatments.

Figure 1 illustrates the XRD patterns of pure and yttrium-doped titanium dioxide systems synthesized through conventional and microwave hydrothermal pathways. The indexed peaks indicate that all analyzed systems exhibit an anatase tetragonal structure with a space group of *I*_41_/*amd*, which closely matches the International Centre for Diffraction Data (ICDD) 9015929 card^30^. The absence of additional crystal phases and shifts in XRD patterns confirms the fabrication of pure anatase structure samples. The larger ion radius of Y^3+^ (0.90 Å) compared to Ti^4+^ (0.61 Å) makes it challenging for Y^3+^ to enter the TiO_2_ lattice^31^. Additionally, the low content and high dispersity of Y species contribute to the absence of extra peaks, such as the oxide form of yttrium^32^. To determine the structural properties of the samples, Rietveld analysis was conducted using FullProf software, and the results for selected systems are presented in Fig. S1 of the Supplementary Materials. The crystallite size (D) was determined using the Scherrer equation (Eq. 1 is presented in the Supplementary Materials) and the corresponding values for all samples are compiled in Table 1. In the context of conventional hydrothermal treatment, no noteworthy distinctions were discerned in the mean crystallite size between the yttrium-doped samples and the pure TiO_2_. However, a marginal augmentation in the average crystallite size was noted with microwave processing. This increase is ascribed to the elevated kinetics inherent in microwave processes, as validated in our earlier research. Irrespective of the hydrothermal treatment method employed, the refined lattice parameters align well with those documented in the literature^33–35^.

**Table 1:** X-ray diffraction, low-temperature N_2_ sorption, and diffuse reflectance spectroscopy results for yttrium-doped TiO_2_ systems.

Sample	*D* (nm)	Lattice parameters		Porous structure parameters			Energy band gap
		Anatase		A_BET_ (m^2^ g^−1^)	V_p_ (cm^3^ g^−1^)	Sp (nm)	E_g_ (eV)
		*a* (Å)	*c* (Å)				
Conventional treatment							
H_TiO_2_	15.6	3.8011	5.5281	178	0.397	3.9	3.2
H_0.25%Y	15.4	3.7838	9.5095	175	0.383	4.4	3.2
H_0.5%Y	15.1	3.7778	9.4832	171	0.372	4.6	3.1
H_1%Y	15.3	3.7951	9.5201	170	0.353	4.8	3.1
H_1.5%Y	15.5	3.7962	9.5143	170	0.348	6.7	3.1
H_2%Y	15.4	3.7935	9.5137	168	0.344	6.8	3.1
Microwave treatment							
M_TiO_2_	15.2	3.7907	9.5050	222	0.376	3.7	3.2
M_0.25%Y	15.5	3.7942	9.5151	214	0.365	4.2	3.2
M_0.5%Y	15.7	3.8081	9.5443	214	0.358	4.2	3.1
M_1%Y	16.1	3.7943	9.5145	212	0.349	4.7	3.1
M_1.5%Y	16.3	3.7891	9.5056	210	0.324	4.9	3.1
M_2%Y	16.5	3.7882	9.5044	209	0.310	4.9	3.1

The parameters related to the porous structure of the synthesized TiO_2_-Y systems are provided in Table 1. Additionally, the N_2_ adsorption–desorption isotherms can be found in Fig. S2 of the Supplementary Materials. The isotherms exhibit type IV characteristics indicating capillary condensation in mesopores and limited uptake at high *p*/*p*_0_ values^36,37^. At low relative pressures, adsorption follows a similar to that of a non-porous adsorbent, undergoing single-layer-multilayer adsorption. Additionally, the materials exhibit an H1 hysteresis loop type, which is typical for porous materials composed of uniform particles arranged in regular arrays with narrow pore size distributions^38^. When comparing the obtained nitrogen adsorption isotherms, no significant differences were observed between the systems synthesized using hydrothermal or microwave treatment. However, the supported yttrium systems exhibit lower BET surface areas compared to bare titanium dioxide. The reduction becomes more pronounced with an increase in yttrium content. Considering that the pore size of anatase ranges between 4–6 nm, it is plausible that yttrium oxide particles are not hosted within the pores but rather adsorbed on the outer surfaces of titanium dioxide. This could lead to the blocking of the porous surface, resulting in a decrease in surface area development and pore volume. Nevertheless, it is crucial to highlight that the utilization of microwave hydrothermal treatment facilitates the enhanced development of the BET surface area.

**Figure 2:**
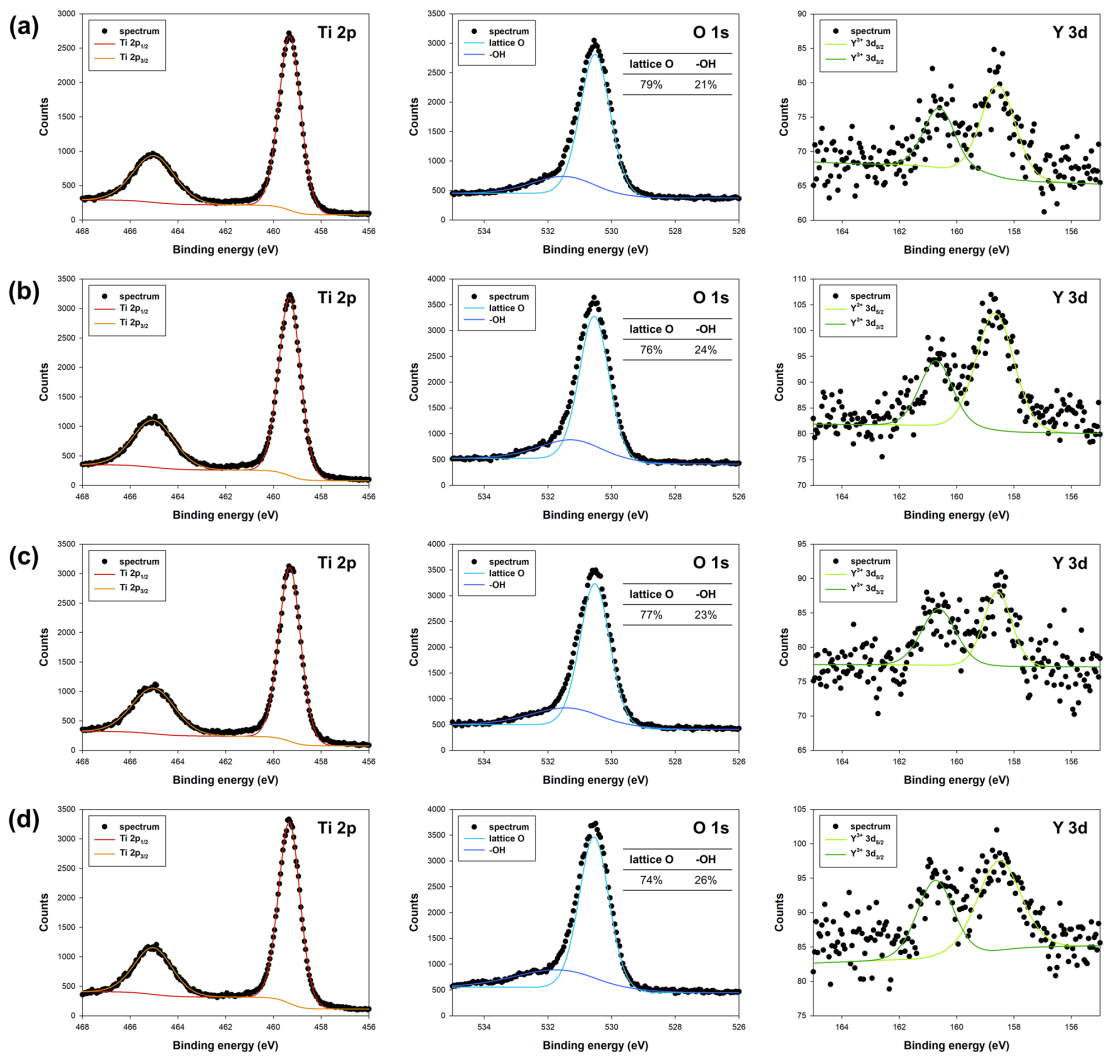
XPS spectra of Ti 2p, O 1 s, and Y 3d regions for (**a**) H_1%Y, (**b**) H_2%Y, (**c**) M_1%Y, and (**d**) M_2%Y.

The X-ray Photoelectron Spectroscopy (XPS) technique was used to investigate the chemical composition of the surface layer (approx. 10 nm depth) in the synthesized TiO_2_-Y systems. Table 2 presents the atomic composition of the detected elements, namely titanium, oxygen, and yttrium. High-resolution (HR) XPS spectra for selected systems are shown in Fig. 2.

**Table 2:** XPS analysis of Ti, O, Y content (at.%) for the synthesized materials.

Sample	Content (at.%)		
	Ti 2p	O 1 s	Y 3d
Conventional treatment			
H_0.25%Y	33.5	66.3	0.2
H_1%Y	31.8	67.4	0.8
H_2%Y	30.6	67.8	1.6
Microwave treatment			
M_0.5%Y	33.6	66.0	0.4
M_1%Y	31.5	67.4	1.1
M_2%Y	29.7	68.2	2.1

It is crucial to highlight a notable difference in the atomic content of yttrium among the acquired TiO_2_-Y systems. The atomic content of yttrium in the microwave-synthesized samples closely aligns with the expected values, whereas in the hydrothermal method, the concentrations are lower than anticipated. Furthermore, the hydrothermal method exhibits lower atomic fractions of oxygen, correlating well with the diminished yttrium content, given that yttrium is present in an oxidized form in the synthesized materials. These findings suggest that the swift microwave method effectively facilitates the doping of TiO_2_ with rare earth metals.

Two distinct peaks were observed in the Ti 2p region with binding energies centered at 458.5 eV and 464.2 eV, corresponding to Ti 2p_3/2_ and Ti 2p_1/2_, respectively^39,40^. The splitting difference of 5.7 eV between these two bands indicates the presence of Ti^4+^ in the sample^41^. The absence of any energy shift in the Ti 2p peaks confirms that yttrium doping occurs only as a surface modification, without incorporating yttrium ions into the anatase lattice structure.

The O 1 s peaks observed at 523.0 eV and 531.5 eV were assigned to lattice oxygen and hydroxyl groups, respectively^42^. It is noteworthy that an increase in yttrium concentration results in a higher oxygen content derived from hydroxyl groups. Additionally, literature reports suggest that these hydroxyl groups can react with photoinduced holes, generating highly oxidizing surface-bound hydroxyl radicals^43^. It is important to mention that, in all analyzed systems, the concentration of hydroxyl groups is higher in materials obtained using microwave hydrothermal treatment.

The binding energy values of Y 3d_5/2_ and Y 3d_3/2_ for the TiO_2_-Y systems are approximately 158.5 eV and 160.5 eV, respectively, with a spin–orbit splitting of 2 eV^44–46^. These results provide evidence that the oxidation state of yttrium ions is predominantly trivalent.

**Figure 3:**
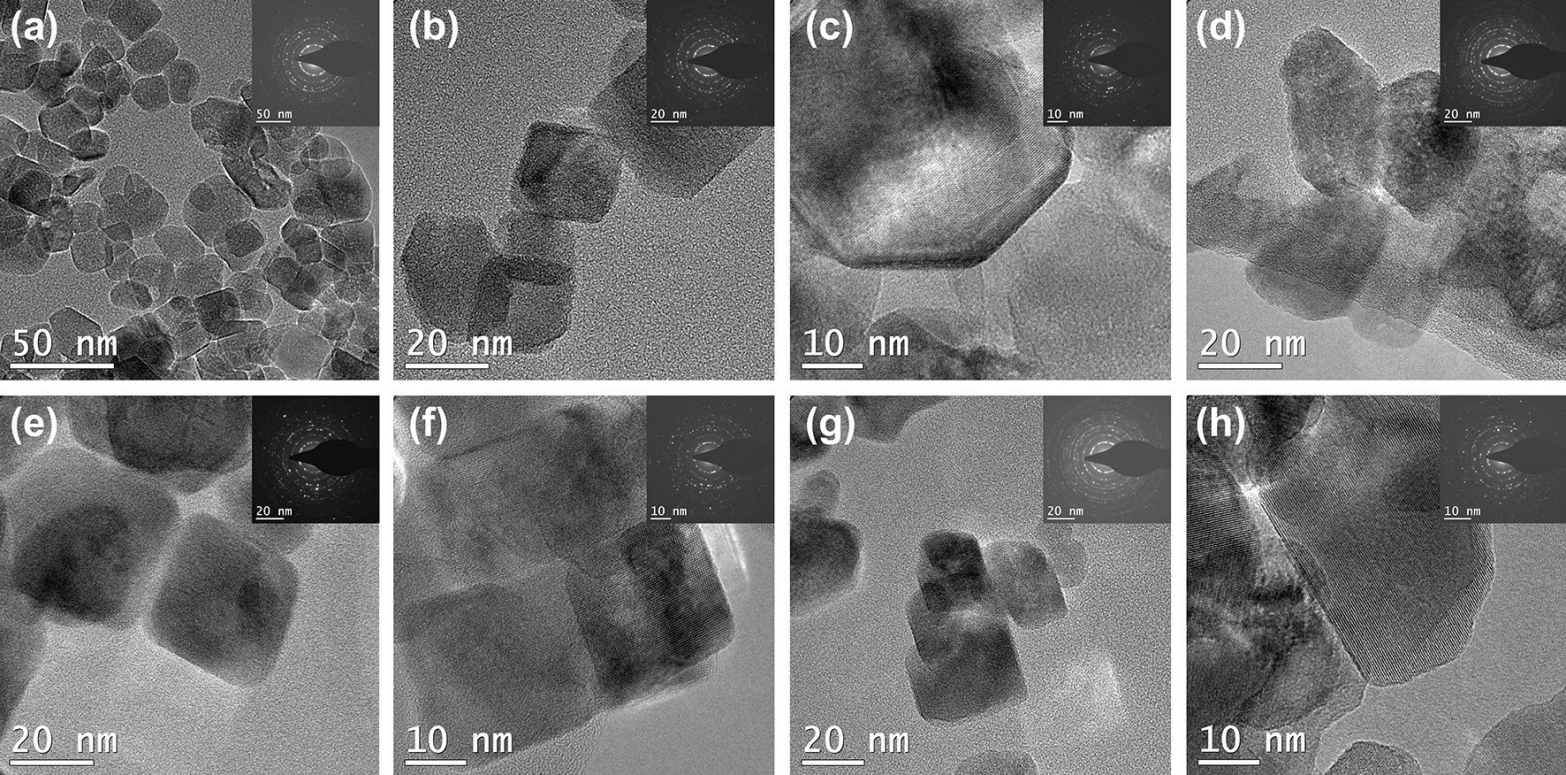
TEM images of: (**a**) H_0.25%Y, (**b**) H_0.5%Y, (**c**) H_1%Y, (d) H_2%Y, (**e**) M_0.25%Y, (**f**) M_0.5%Y, (**g**) M_1%Y, and (M) M_2%Y. SAED patterns are shown in the inset.

A TEM study was conducted to explore the impact of yttrium doping on the morphology of anatase, as illustrated in Fig. 3. The average particle size of the nanoparticles (NPs) is 20–25 nm, displaying a regular shape characteristic of bare anatase. However, unlike the regular structure observed in samples with lower yttrium content (e.g., 0.25% and 0.5 wt.% Y), the presence of 1% and 2% wt. Y results in NPs with more irregular particle edges. This irregularity is particularly pronounced in materials obtained through the microwave pathway. These irregular structures were also described by Bingxin Zhao et al.^34^, who highlighted their role in facilitating the diffusion of photoinduced electrons. The selected area electron diffraction (SAED) patterns, shown in the insets for all analyzed systems, clearly indicate the presence of polycrystalline circles in the anatase phase^47,48^. Figure S3 (see Supplementary Materials) presents the TEM d-spacing profile, demonstrating that the (101) planes did not experience expansion due to yttrium doping. This finding aligns well with the calculated interplanar distance (101) based on the diffraction angle, confirming that yttrium ions have not entered the TiO_2_ lattice^49^. Additionally, it should be noted that, in the case of TiO_2_-Y systems, no significant changes in sample morphology were observed based on the type of hydrothermal treatment used, whether conventional or microwave.

The EDX results and elemental mapping, depicted in Fig. S4 in the Supplementary Materials, highlight the presence of three main elements in the synthesized systems: titanium, oxygen, and yttrium. Importantly, the distribution of yttrium is uniform across the entire sample area in all analyzed materials. Furthermore, it is noteworthy that the EDX results (refer to Table S1 in the Supplementary Materials) corroborate the earlier conclusions drawn from the XPS analysis. They confirm that the microwave method is more effective than the hydrothermal method in the yttrium doping process, resulting in a higher weight percentage of yttrium in systems obtained by the microwave route.

### Photocatalytic ability

In order to investigate the optical response of TiO_2_-Y systems, UV–Visible diffuse reflectance spectra were conducted. The DRS spectra of Y-doped TiO_2_ systems synthesized through various hydrothermal routes are shown in Fig. S5 (refer to Supplementary Materials). Compared to bare TiO_2_, the optical absorption edges of TiO_2_-Y systems exhibited a red shift, particularly in materials with lower weight percentages of yttrium ions. This shift in the optical absorption band can be attributed to the presence of Y ions on the surface of TiO_2_, potentially causing a charge imbalance transition between the Y 3d electrons and the conduction or valence electrons of the pure TiO_2_. The creation of dopant levels near the conduction band can explain the red shift in the band gap. To determine the optical band gaps, the diffuse reflectance (R) of both the undoped and TiO_2_-Y systems can be correlated to the Kubelka–Munk function. The calculated band gaps were approximately 3.2 eV for bare TiO_2_ and 3.1 eV for yttrium-doped TiO_2_ systems. Niu et al.^50^ previously reported a red shift in the band gap of Y-doped TiO_2_ nanoparticles, attributed to charge transfer between the f electrons of Y^3+^ and the TiO_2_ conduction or valence band, as observed through diffuse reflectance studies. Muktaridha et al.^51^ also reported a blue shift in the band gap of Y-doped systems using UV–Visible absorption studies. This blue shift was explained by the occupied states of Ti 3d and the up-shift of the Fermi level. The reason for these blue shifts was the gradual movement of the conduction band due to dopant incorporation, which is not observed in the present study.

**Figure 4:**
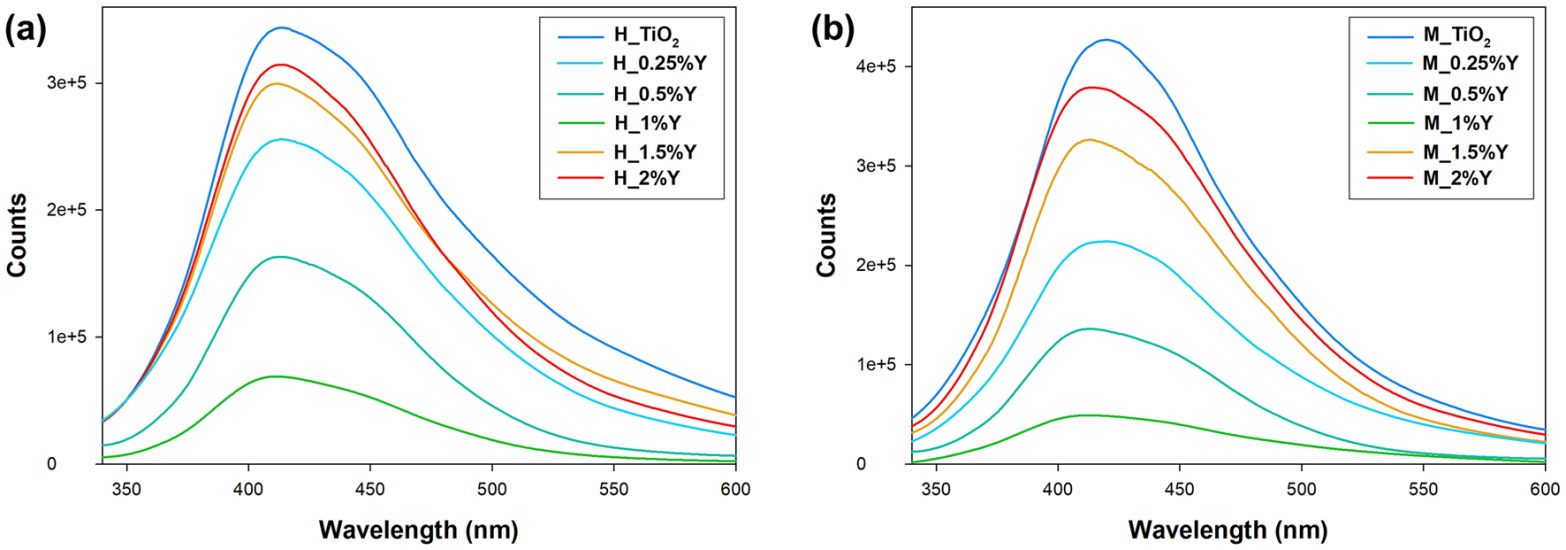
The PL spectra for TiO_2_-Y systems synthesized by (**a**) conventional and (**b**) microwave treatments.

The room-temperature photoluminescence (PL) (Fig. 4) spectra exhibit a distinctive broad and intense emission band around 450 nm, characteristic of pure TiO_2_^52^. The shape of the spectra indicates that the presence of yttrium does not introduce any new photoluminescence features. Previous studies on titanium dioxide emissions have identified two primary peaks at approximately 396 nm and 462 nm, corresponding to energy levels of 3.13 eV and 2.68 eV, respectively^53,54^. However, a broader luminescence band has also been observed, resulting from merging these two narrow peaks. These peaks are associated with the bandgap and charge-transfer transitions from Ti^3+^ to oxygen anions in a TiO_6_^8−^ complex. Interestingly, regardless of the materials analyzed, there is a noticeable emission suppression in TiO_2_-Y systems, indicating hindered recombination of photogenerated holes and electrons^54,55^. The presence of yttrium ions introduces additional electron transport pathways, facilitating faster separation and efficient transport of photogenerated charge carriers. This enhanced charge carrier mobility contributes to reduced recombination rates and suppressed photoluminescence emission in yttrium-doped TiO_2_. In yttrium-doped systems, the introduction of more traps into TiO_2_ and the trapping of additional photoelectrons prevent recombination, resulting in reduced photoluminescence emission intensity. However, it is important to note that an increase in luminescence has been observed with higher yttrium content. This effect can be attributed to the modification of charge carrier dynamics by the high yttrium concentration, leading to a more significant population of excited states^56,57^. As a result, there is a higher emission intensity upon excitation. Furthermore, the choice of hydrothermal synthesis route impacts the efficiency of photoluminescence. The conventional method yields a higher luminescence yield, likely due to the lower yttrium content in these materials. On the other hand, as previously mentioned, the microwave method allows for effective doping of TiO_2_ with rare earth metals, leading to better quenching of luminescence and, consequently, more efficient separation of charge carriers.

**Figure 5:**
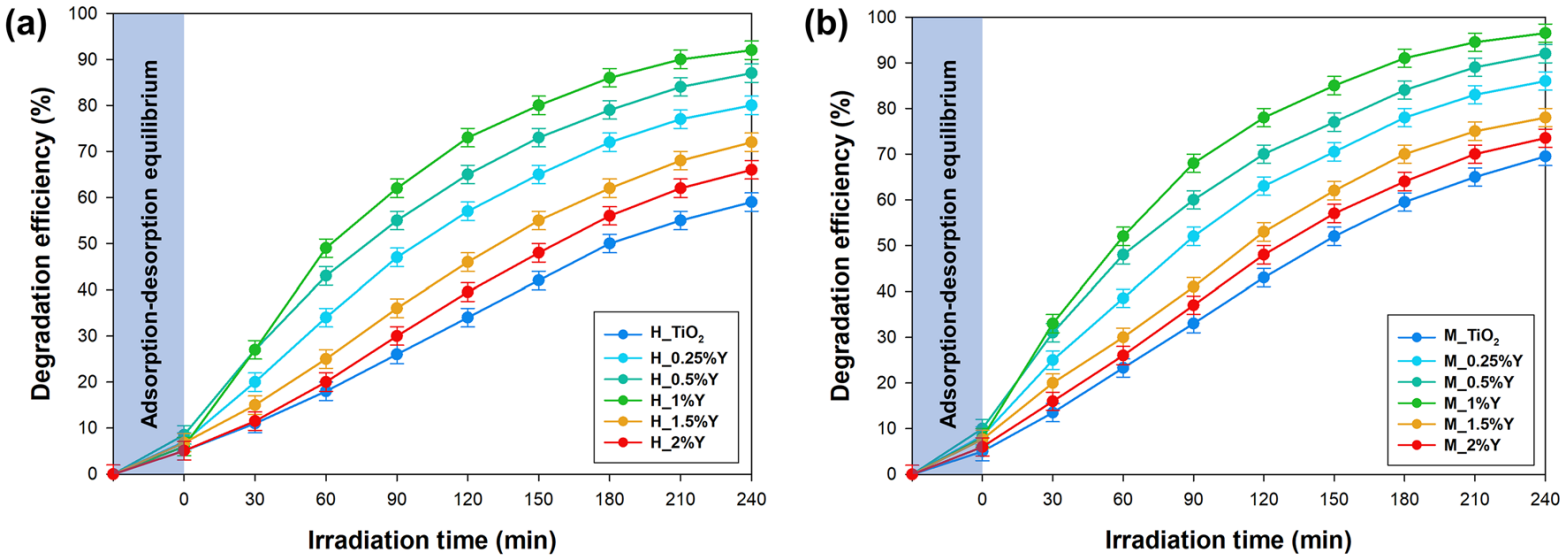
The efficiency of carbamazepine photo-oxidation using TiO_2_-Y systems synthesized by (**a**) conventional and (**b**) microwave treatments.

The crucial point of the research was to determine the effectiveness of the fabricated TiO_2_-Y systems in removing harmful carbamazepine waste through the photooxidation process. The collected degradation curves are presented in Fig. 5.

Under UV-LED light irradiation, CBZ degradation in the presence of TiO_2_ was at a low level, amounting to 60% and 70% of the efficiency for conventional and microwave processing, respectively. Doping bare TiO_2_ with small amounts of yttrium ions (0.25–1 wt.% Y) significantly improved photocatalytic activity. Almost all of the CBZ was removed (91% and 96% for conventional and microwave treatment, respectively) when the yttrium concentration was 1 wt.%. This enhanced photocatalytic activity was attributed to the efficient junctions between yttrium ions and the surface of TiO_2_. The interaction of dopant ions with the semiconductor's surface increased light absorbance and boosted carrier transfer. However, excessive yttrium dopants may act as recombination centers for carriers and hinder light absorption^58,59^. Consequently, the photo-oxidation efficiency of CBZ decreases to 66% and 73% for conventional and microwave treatment, respectively, when the systems contain 2 wt.% of yttrium.

It should be noted that changing the hydrothermal treatment method impacts the photocatalytic performance of yttrium-doped TiO_2_ systems. Regardless of the specific system analyzed, samples produced using the microwave method exhibited increased efficiency in removing CBZ. Several factors contribute to this observation. While the doping process did not alter the electronic structure of TiO_2_, it did enhance the absorption of UV radiation, as indicated by the calculation of bandgap energy. Moreover, the presented photoluminescence spectra demonstrated improved luminescence quenching for systems obtained through the microwave pathway, indicating a more effective transfer of electron–hole charge carriers. Additionally, it is worth emphasizing that materials prepared using microwave radiation show higher surface area development, a critical parameter in the photo-oxidation process. Furthermore, microwave processing resulted in a larger average crystallite size and facilitated the attainment of desired levels of yttrium on the surface of bare TiO_2_. Altogether, these factors collectively contributed to the observed enhancement in the photocatalytic performance of TiO_2_-Y systems synthesized using the microwave method.

The revised Langmuir–Hinshelwood model provides a suitable framework for elucidating the degradation mechanism, aligning with the surface reaction concept frequently examined in existing literature^60–62^. To determine the rate constant parameter, the sorption process was intentionally omitted from the calculations. In accordance with Eqs. (2) and (3), the apparent values of parameter k_1_ for each catalyst were computed by analyzing the slope of the ln(C_t_/C_0_) vs. time plot. The calculated values are presented in Table S2, available in the Supplementary Materials for comparison. Consistent with previously presented results, among a series of materials, the M_1%Y catalyst exhibits the highest reaction rate constant at 0.0135 min^−1^. In contrast, the material obtained through the conventional hydrothermal method showed a consistent reaction rate of 0.0108 min^−1^ – H_1%Y. Significantly, regardless of the series of materials analyzed, transitioning from the microwave treatment method to the conventional hydrothermal technique consistently results in a decrease in the reaction rate constant. This reduction can be primarily attributed to an increased recombination rate and diminished absorption of UV light.

Table 3 shows the current state of knowledge in the field of photocatalytic degradation of carbamazepine.

**Table 3:** Summary of literature data on the degradation of carbamazepine.

Sample	CBZ concentration (mg/dm^3^)	Amount of photocatalyst (g/dm^3^)	Degradation efficiency (%)	Irradiation time (min)	Parameters of the light source	Refs.
M_1%Y	20	1	95	240	UV-LED (395 nm)	This work
g-C_3_N_4_/TiO_2_	10	1	72	360	UV-A lamp 24 W	^63^
Fe_3_O_4_@SiO_2_/d-TiO_2_-Pt + H_2_O_2_	14	0.5	95	120	Xe-lamp 300W	^64^
P25	10	1	70	120	Xe-lamp 1000W	^65^
Ag_3_PO_4_/GO	5	0.5	99	120	PCX50C Discover (420 nm)	^66^
TiO_2_	10	1.5	99	120	UV-A (365 nm)	^67^
graphene oxide/TiO_2_	10	0.5	99	90	Hg-lamp (125W)	^68^
TiO_2_/Ti_3_C_2_T_x_	5	0.5	98	180	Solar simulator	^69^

The presented data underscores the importance of addressing the challenge of carbamazepine removal through photocatalysis, as evidenced by extensive research within the scientific community. A thorough analysis of existing scientific literature reveals a common reliance on photocatalytic water purification systems with ultraviolet light sources. The duration of the photocatalytic process is intricately linked to factors such as CBZ concentration and the type and quantity of the photocatalyst used. It is crucial to note that certain referenced studies still employ gas discharge lamps, a practice inconsistent with the principles of green chemistry and sustainable development. In this context, our study's results, based on the implementation of an LED solution, seem to align more closely with the expectations of the scientific community. The achieved photoactivity with the M_1%Y catalyst notably corresponds with values documented in the prevailing scientific literature. Furthermore, the adoption of the LED system has the potential to reduce electricity demand, a factor we previously addressed in our research^70,71^.

For the reusability test, the photocatalysts H_1%Y and M_1%Y were chosen. Five consecutive cycles of CBZ photo-oxidation were carried out to evaluate their photocatalytic reusability, as shown in Fig. S6 of the Supplementary Materials. After each cycle, the photocatalyst was separated from the reaction suspension through filtration and reused without any additional treatment. The results showed that the efficiency of photocatalytic degradation decreased by approximately 10% after the 5th cycle compared to the 1st cycle. This slight decline in activity after each irradiation cycle could be attributed to the loss of photocatalysts during the separation process.

The FTIR spectra of the TiO_2_-Y samples (Fig. S7, Supplementary Materials) showed no significant difference between the samples before and after the CBZ photo-oxidation process. Prior to irradiation, the spectra exhibited stretching vibrations of Ti–O bonds (800–900 cm^−1^)^72^ and surface hydroxyl groups (1625 cm^−1^ and 3430 cm^−1^)^73,74^ in the mentioned systems. However, no additional bands were observed after the CBZ photo-oxidation process in the TiO_2_-Y systems. The only noticeable change was a decrease in the intensity of bands originating from the surface hydroxyl groups^75,76^. These spectra confirm that there is minimal alteration to the surface of the analyzed photocatalysts, which is consistent with the results obtained from the previous reusability tests.

Figure S8 (see Supplementary Materials) shows the data obtained by introducing scavengers into the CBZ solution to explore the degradation mechanism in yttrium-doped TiO_2_ systems. These scavengers were intended to capture the photo-generated electrons, holes, and primary reactive oxygen species such as ^*^OH and ^*^O_2_^−^. The efficiency of CBZ photooxidation decreased by approximately 20% upon the introduction of ammonium oxalate and silver nitrate, which acted as scavengers for electrons and holes. The reason for only a slight decrease in CBZ removal efficiency can be due to the enhanced separation of charge carriers facilitated by the presence of yttrium ions on the TiO_2_ surface^77,78^. This effect was confirmed by analyzing the photoluminescence spectra. Furthermore, the addition of *tert*-butanol, a scavenger for hydroxyl radicals (^*^OH), significantly decreased the photodegradation efficiency, reducing it by approximately 51% for the analyzed materials. This suggests that ^*^OH is an active species involved in the photo-oxidation process. On the other hand, the scavenging of superoxide radical anions (^*^O_2_^−^) considerably impacted the efficiency of CBZ removal. In the case of the analyzed samples, it led to a decrease in efficiency of 26% and 20% for M_1%Y and H_1%Y, respectively. This indicates that ^*^O_2_^−^ plays a crucial role in the photocatalytic degradation mechanism. Based on these findings, it can be inferred that the mechanisms occurring in the aquatic environment are primarily governed by the generation of reactive oxygen species (ROS), including hydroxyl radicals and superoxide radical anions^79,80^. The major steps in the photocatalytic mechanism are illustrated in the equations below.

4 $$TiO_{2} - Y\;photocatalyst + hv \to e^{-} + h^{ + }$$

5 $$e^{ - } + O_{2} \to ^{*}O_{2}^{-}$$

6 $$h^{ + } + H_{2} O/OH^{ - } \to H^{ + } + OH^{*}$$

7 $$^{*}OH + carbamazepine \to degradation\;products$$

8 $$^{*}O_{2}^{-} + carbamazepine \to deggradation\;products$$

The products of CBZ photodegradation were detected using the MS technique and identified based on their mass spectra. The resulting mass spectra are shown in Table S3 in the Supplementary Materials. Figure S9 (see in Supplementary Materials) illustrates the mechanism of by-product formation, which is further discussed compared to existing literature. During the photocatalytic degradation of CBZ, two primary pathways are observed. Hydroxyl radical dot radicals, generated during the photocatalytic process, attack the aromatic ring of CBZ, resulting in the formation of C1 (monohydroxy CBZ, *m/z* = 253)^81,82^. The subsequent addition of another hydroxyl radical leads to the formation of C2 (dihydroxy CBZ, *m/z* = 265)^83^. The by-product at C3 (*m/z* = 238) is formed through a combination of ring contraction and reduction (-CONH_2_) of (1,1(1-aminovinyl)-10,10a-dihydroacridine-9-carbaldehyde)^83^. Further degradation of the intermediate product C3 via oxidation yields C4 (acridine, *m/z* = 180), while the transformation products C5 (2-hydroxy acridine-9-carboxaldehyde, *m/z* = 224) and C6 (formaldehyde-acridine (1/1), *m/z* = 195) are also likely to be present^84^. Finally, these intermediate products undergo an additional ring-opening reaction, resulting in the production of acetic acid, which can be mineralized into CO_2_ and H_2_O through oxidation.

## Conclusions

The findings presented in this study underscore the remarkable enhancement of yttrium-doped TiO_2_'s photocatalytic activity for CBZ degradation under UV-LED light irradiation. The introduction of small quantities of yttrium ions (0.25–1 wt.%) resulted in a substantial improvement in photocatalytic efficiency, culminating in nearly complete CBZ removal at a yttrium concentration of 1 wt.%. This augmented performance can be attributed to the formation of efficient junctions between yttrium ions and the TiO_2_ surface, amplifying light absorption and carrier transfer processes. Notably, the choice of hydrothermal treatment method played a pivotal role in influencing the photocatalytic efficacy of yttrium-doped TiO_2_ systems. Microwave-assisted hydrothermal synthesis exhibited a superior capacity for CBZ removal when compared to conventional hydrothermal techniques. This superiority arises from several factors, including heightened light absorption, more efficient charge carrier transfer, expanded surface area, and increased average crystallite size achieved through microwave processing.

Furthermore, the examination of scavengers unveiled the involvement of reactive oxygen species (ROS), including hydroxyl radicals (^*^OH) and superoxide radical anions (^*^O^2−^) in the photocatalytic degradation mechanism. The introduction of scavengers led to a reduction in the efficiency of CBZ removal, underscoring the significance of these species in the degradation process. Collectively, this study offers valuable insights into the utilization of microwave technology in yttrium-doped TiO_2_ fabrication, shedding light on its substantial influence on photocatalytic performance. It highlights the potential of microwaves as an innovative and eco-friendly synthesis technique, exemplified through the enhanced photocatalysis of yttrium-doped TiO_2_. However, it is essential to acknowledge that a comprehensive understanding of the underlying mechanisms remains a critical stepping stone towards fully harnessing the environmental and economic benefits of this approach.

### Supplementary Information

Supplementary Information.

## Data Availability

The data that support the findings of this research are available from the corresponding author upon reasonable request.
