# Supplementary material for: Unraveling the impact of microwave-assisted techniques in the fabrication of yttrium-doped TiO2 photocatalyst
Source: Sci Rep. 2024 Jan 2;14:262. doi: 10.1038/s41598-023-51078-0 (PMC10761958; doi:10.1038/s41598-023-51078-0)
Supplement: Supplementary file 1 — Supplementary Information. [file 41598_2023_51078_MOESM1_ESM.docx]

**Unraveling the impact of microwave-assisted techniques in the fabrication of yttrium-doped TiO_2_ photocatalyst**

Adam Kubiak^*^, Michał Cegłowski

*Adam Mickiewicz University, Poznan, Faculty of Chemistry, Uniwersytetu Poznanskiego 8, PL-61614 Poznan, Poland*

**Corresponding author: adam.kubiak@amu.edu.pl; Tel.: +48 61 829 17 21*

**Materials and method**

The crystal structure was determined using the X-ray diffraction technique. The Rigaku Miniflex 600 instrument from Rigaku, Japan, operating with Cu Kα radiation (λ=1.5418 Å), was employed to obtain diffraction patterns spanning an angular range of 20-80°. To analyze the patterns, the Rietveld method was employed via the Fullprof software. The crystallite size of the composites synthesized along the vertical direction of the lattice plane was calculated using Scherrer's equation (Eq.1). The quantification of phase composition with standard deviation was performed utilizing the Reference Intensity Ratio (RIR) method, considering the most intense independent peak of each phase.

| $D=\frac{K\lambda}{\beta cos\theta}$ | *(1)* |
| --- | --- |

where:

*D* – average crystallite size (nm); *K* – Scherrer constant (0.891); *λ* – X-ray wavelength (λ=1.5406 Å); *β* – line broadening at half the maximum intensity (FWHM), *θ* – Bragg angle (degree).

The surface area, pore volume, and pore diameter were characterized through the low-temperature nitrogen sorption method, employing the 3FLEX porosimeter from Micromeritics Instrument Co., USA. Prior to the measurements, the materials were subjected to degassing at 120 °C for 4 h. The surface area was determined using the multipoint BET method, analyzing the adsorption data within a relative pressure (*p/p_0_*) range of 0.05-0.30.

X-ray Photoelectron Spectroscopy (XPS) experiments were carried out using a Specs UHV spectrometer from SPECS, Germany, equipped with a charge neutralizer. The rectification of binding energies was achieved using the C 1s peak at 284.8 eV as a reference.

The morphology of the TiO_2_-Y systems was investigated utilizing a transmission electron microscope (TEM, HT7700, Hitachi, Japan) operating in high contrast and high-resolution mode.

The elemental composition of the samples was analyzed using a scanning electron microscope (SEM) 1430 VP by LEO Electron Microscopy Ltd, England. Energy dispersive X-ray spectrometry (EDX) was conducted using a Quantax 200 X-ray spectrometer with an XFlash 4010 detector, by Bruker AXS.

Diffuse reflectance spectroscopy (DRS) was employed to evaluate the light absorption properties of the oxide materials. The measurements were conducted using a Thermo Scientific Evolution 220 spectrophotometer from Waltham, USA, equipped with a PIN-757 integrating sphere, while BaSO_4_ was utilized as the reference material. The bandgap energy of the samples was computed from the plot of (F(R)·E)^0.5^ against E, where E represents the photon energy, and F(R) denotes the Kubelka-Munk function proportional to the radiation absorption.

For photoluminescence (PL) analysis, a spectrofluorometer (Fluorolog version-3 Horiba, Japan) was utilized, with a 450 W high-pressure xenon arc lamp serving as the excitation source. The photoluminescence excitation was set at a wavelength of 330 nm, and both excitation and emission spectra were recorded at room temperature, with a spectral resolution of 2 nm and a slit width of 2 mm.


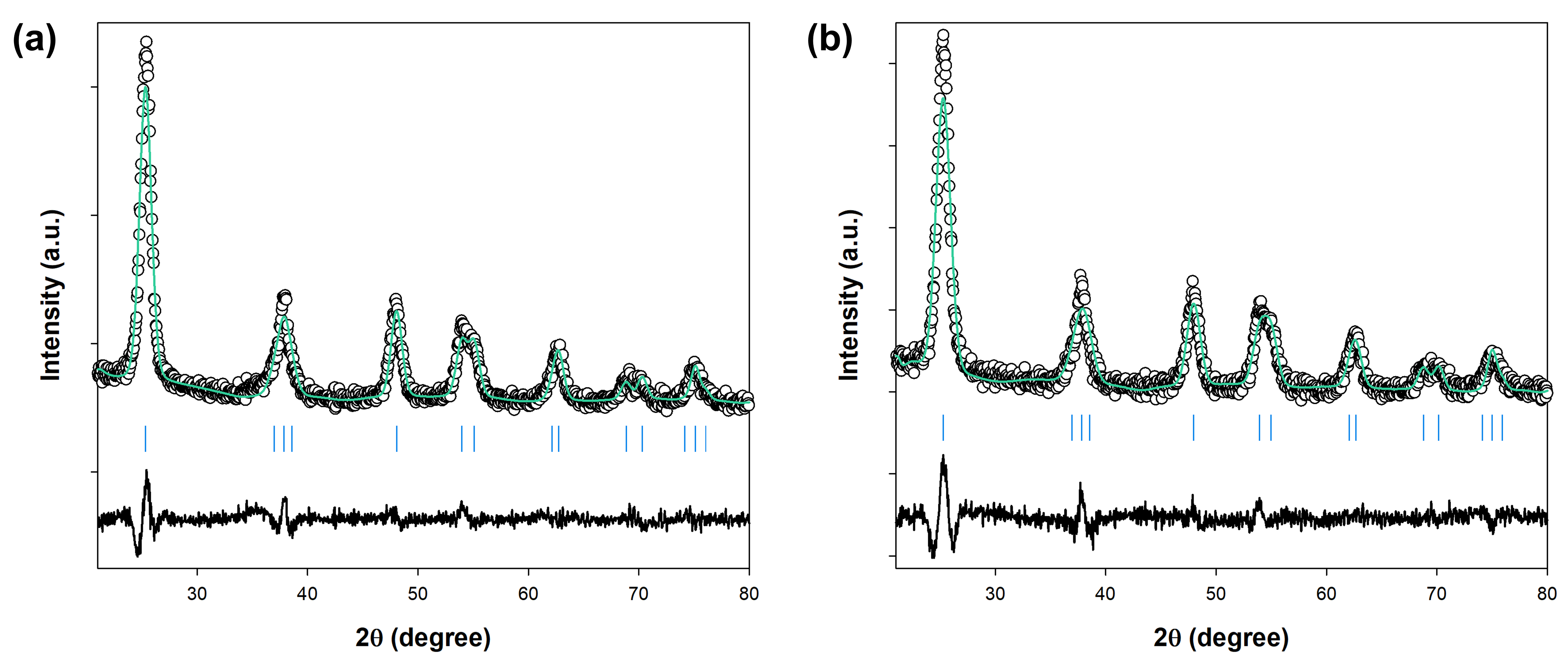


**Fig. S1.** Example of Rietveld refinement for (a) H_1%Y, and (b) M_1%Y.


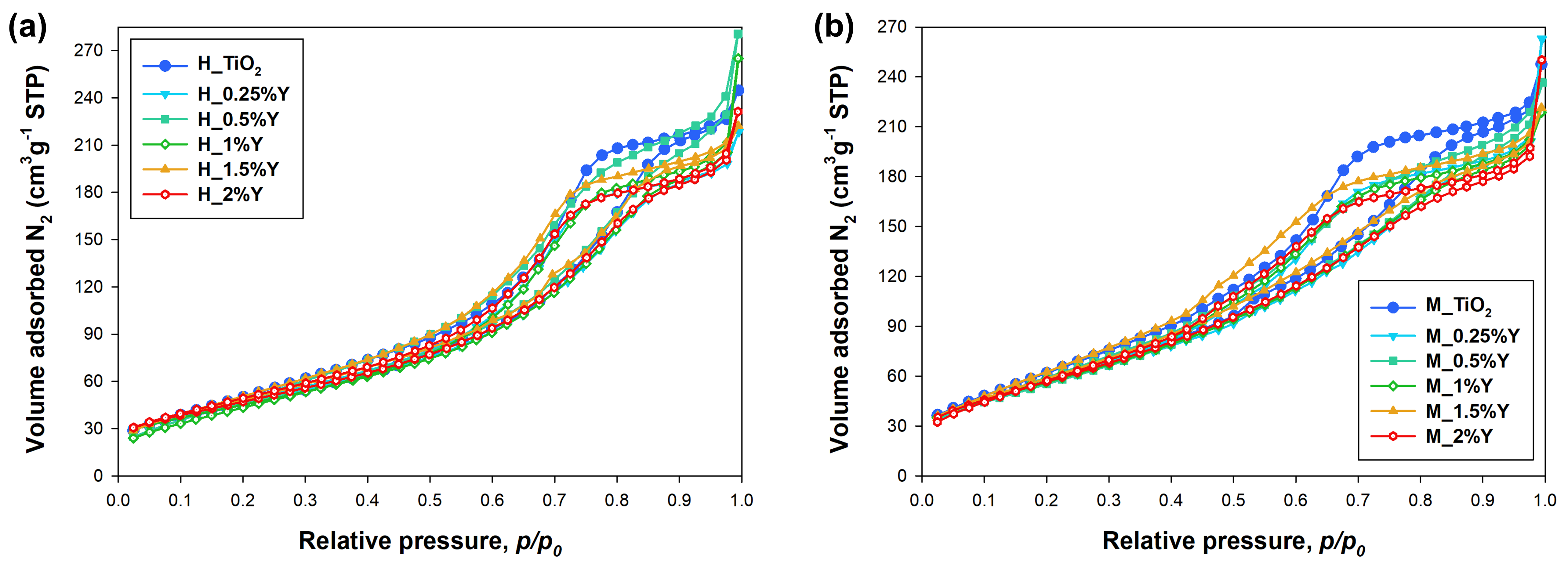


**Fig. S2.** N_2_ adsorption/desorption isotherms of materials synthesized by (a) conventional, and (b) microwave hydrothermal treatment.


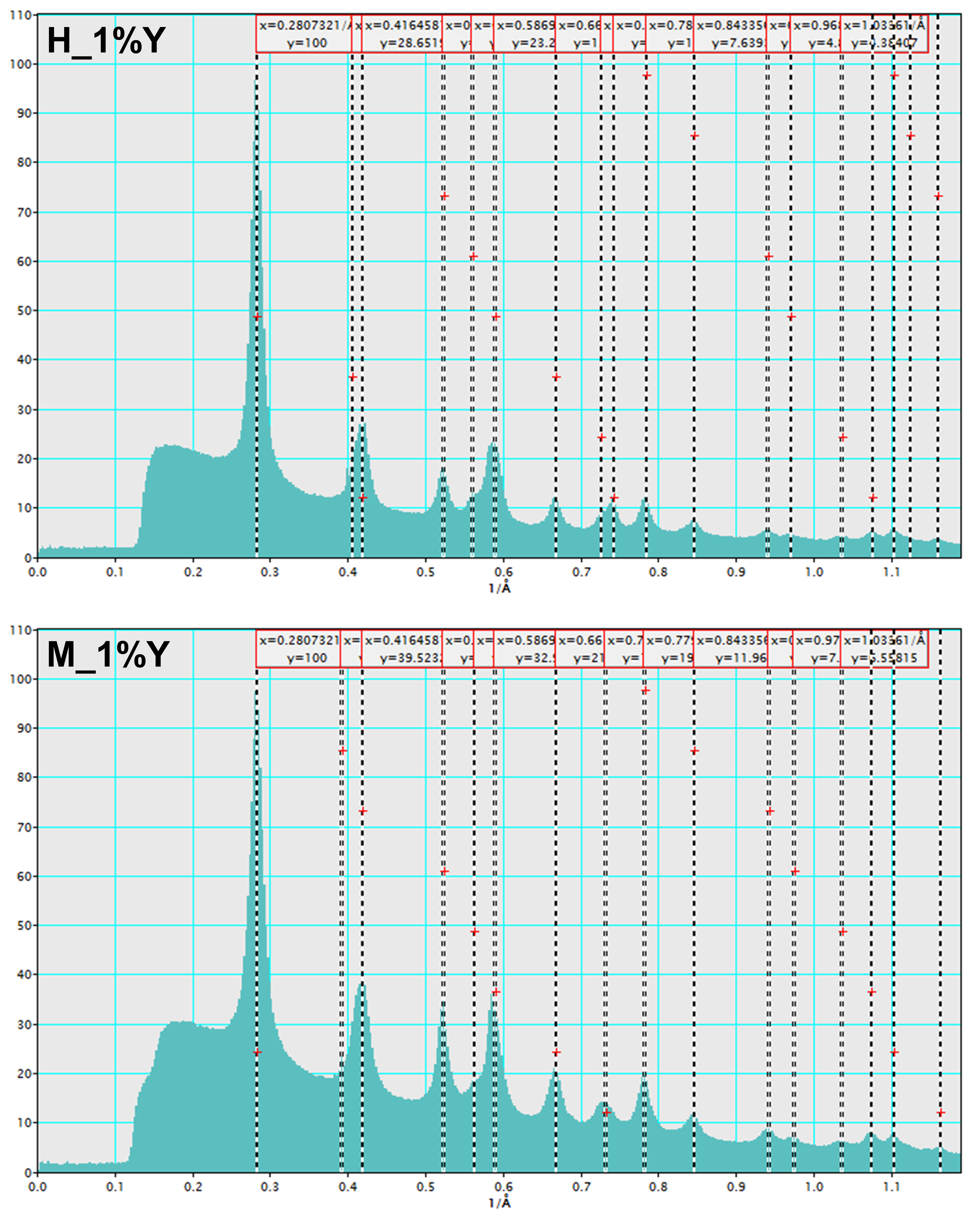


**Fig. S3.** TEM d-spacing profile for selected TiO_2_-Y systems.


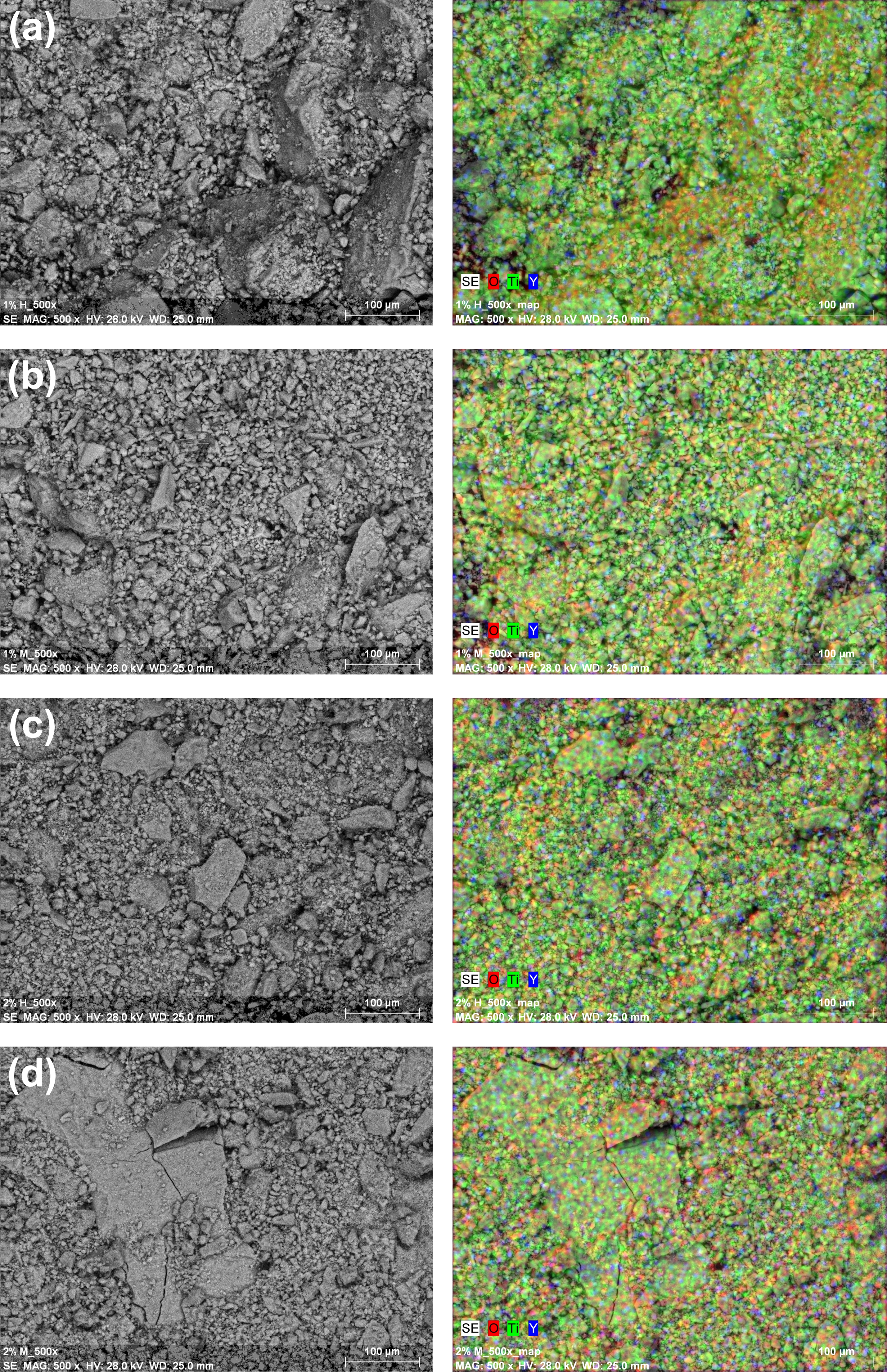


**Fig. S4.** The SEM images and EDX mapping for: (a) H_1%Y, (b) M_1%Y, (c) H_2%Y, and (d) M_2%Y.

**Table S1.** Surface composition of selected TiO_2_-Y systems based on EDX measurement.

| Sample | Titanium (wt. %) | Oxygen (wt. %) | Yttrium (wt. %) |
| --- | --- | --- | --- |
| H_1%Y | 60.92 | 38.30 | 0.78 |
| M_1%Y | 60.60 | 38.15 | 1.25 |
| H_2%Y | 61.46 | 36.72 | 1.82 |
| M_2%Y | 61.13 | 36.70 | 2.17 |


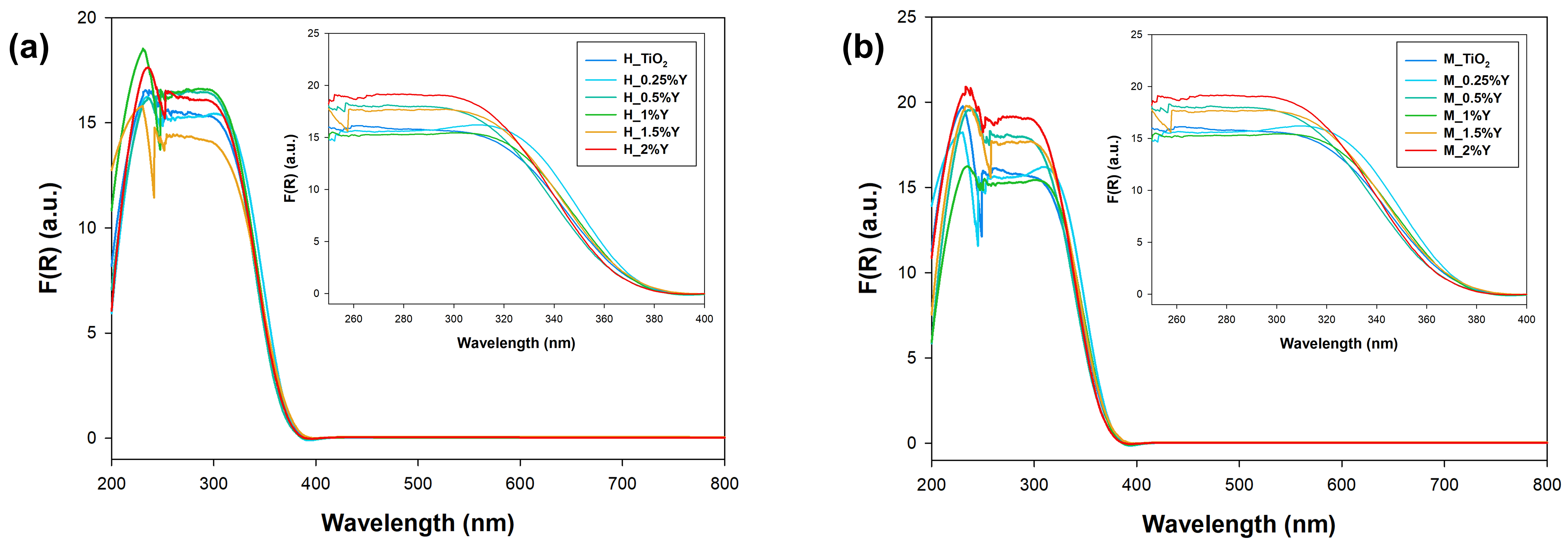


**Fig. S5.** DRS spectra of materials synthesized by (a) conventional, and (b) microwave hydrothermal treatment.

**Table S2.** The calculated kinetic parameters for the photochemical degradation of CBZ.

| Sample | k_1_ (1/min) | (R^2^) |
| --- | --- | --- |
| conventional treatment | | |
| H_TiO_2_ | 0.0037 | 0.998 |
| H_0.25%Y | 0.0069 | 0.997 |
| H_0.5%Y | 0.0087 | 0.997 |
| H_1%Y | 0.0108 | 0.998 |
| H_1.5%Y | 0.0053 | 0.997 |
| H_2%Y | 0.0045 | 0.998 |
| microwave treatment | | |
| M_TiO_2_ | 0.0049 | 0.999 |
| M_0.25%Y | 0.0083 | 0.997 |
| M_0.5%Y | 0.0103 | 0.997 |
| M_1%Y | 0.0135 | 0.998 |
| M_1.5%Y | 0.0064 | 0.998 |
| M_2%Y | 0.0056 | 0.996 |


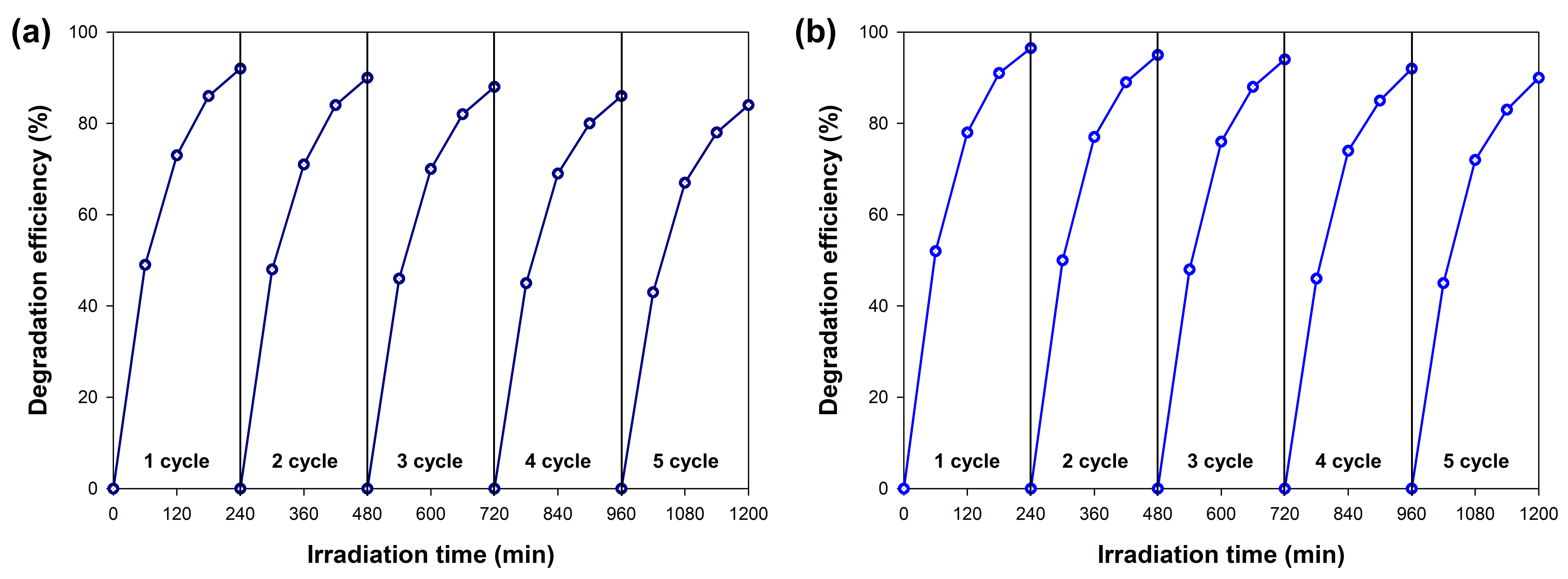


**Fig. S6.** Efficiency of SMX photo-oxidation in the presence of (a) H_1%Y and (b) M_1%Y photocatalysts measured over five successive cycles.


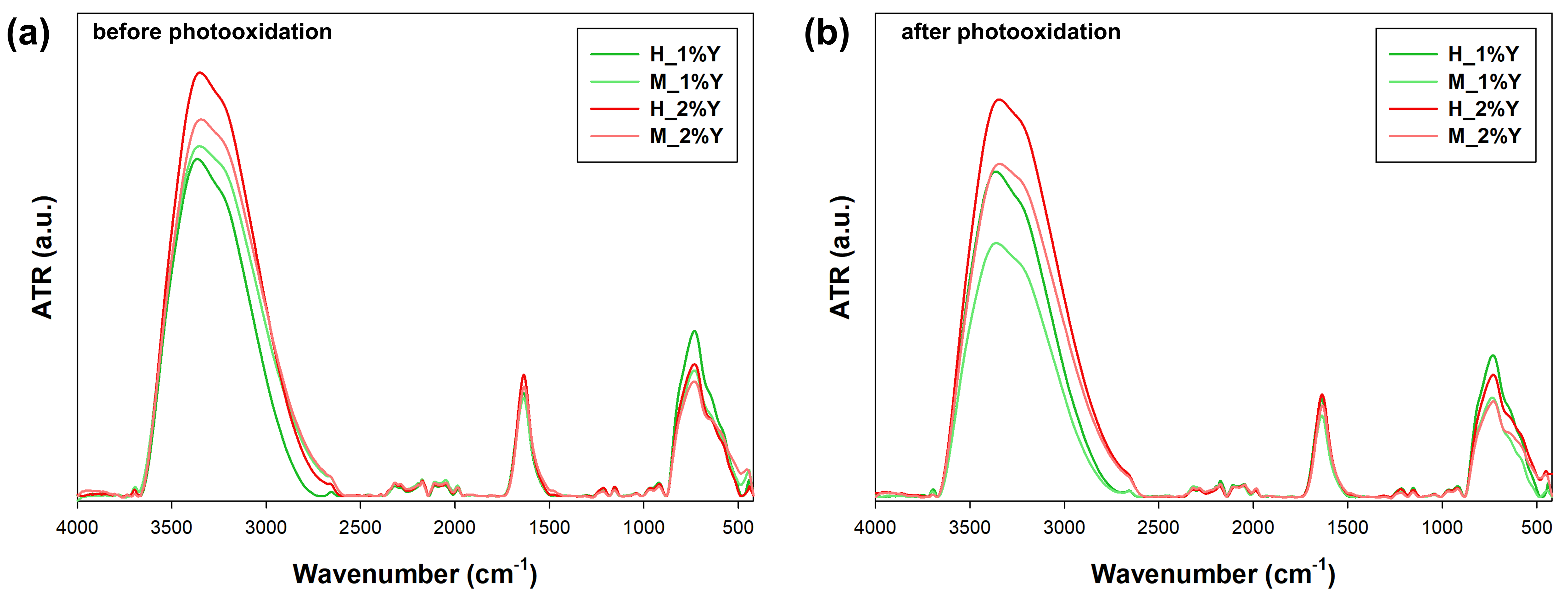


**Fig. S7.** The FTIR spectra for selected TiO_2_-Y samples (a) before and (b) after photocatalysis.


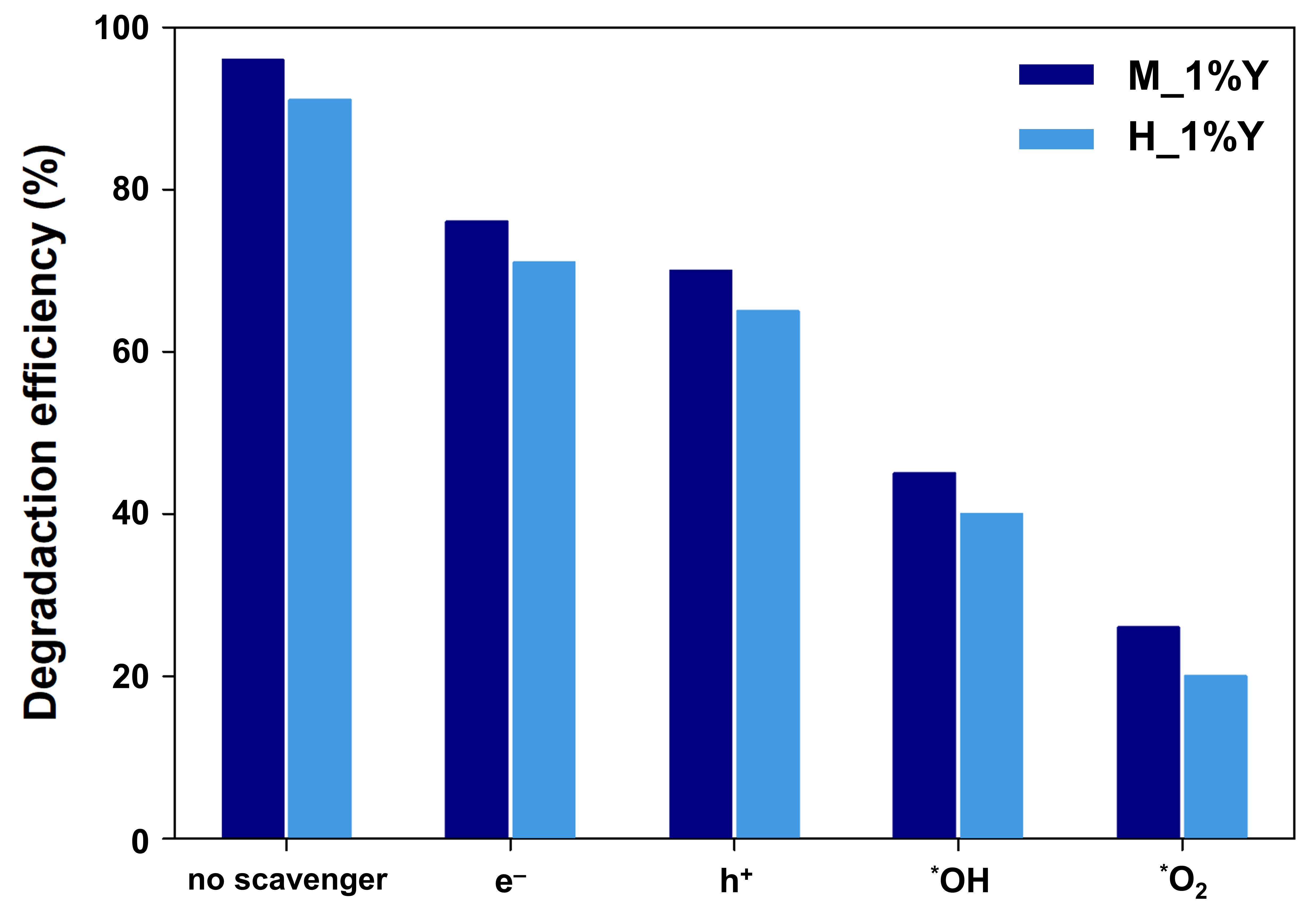


**Fig. S8.** The degradation efficiency of CBZ in the presence of radical scavengers.

**Table S3.** The mass spectra of the generated ions during the photo-oxidation of CBZ using TiO_2_-Y photocatalysts

| CBZ | *positive mode* |  |
| --- | --- | --- |
|  | *negative mode* |  |
| H_1%Y  (2h) | *positive mode* |  |
|  | *negative mode* |  |
| M_1%Y  (2h) | *positive mode* |  |
|  | *negative mode* |  |


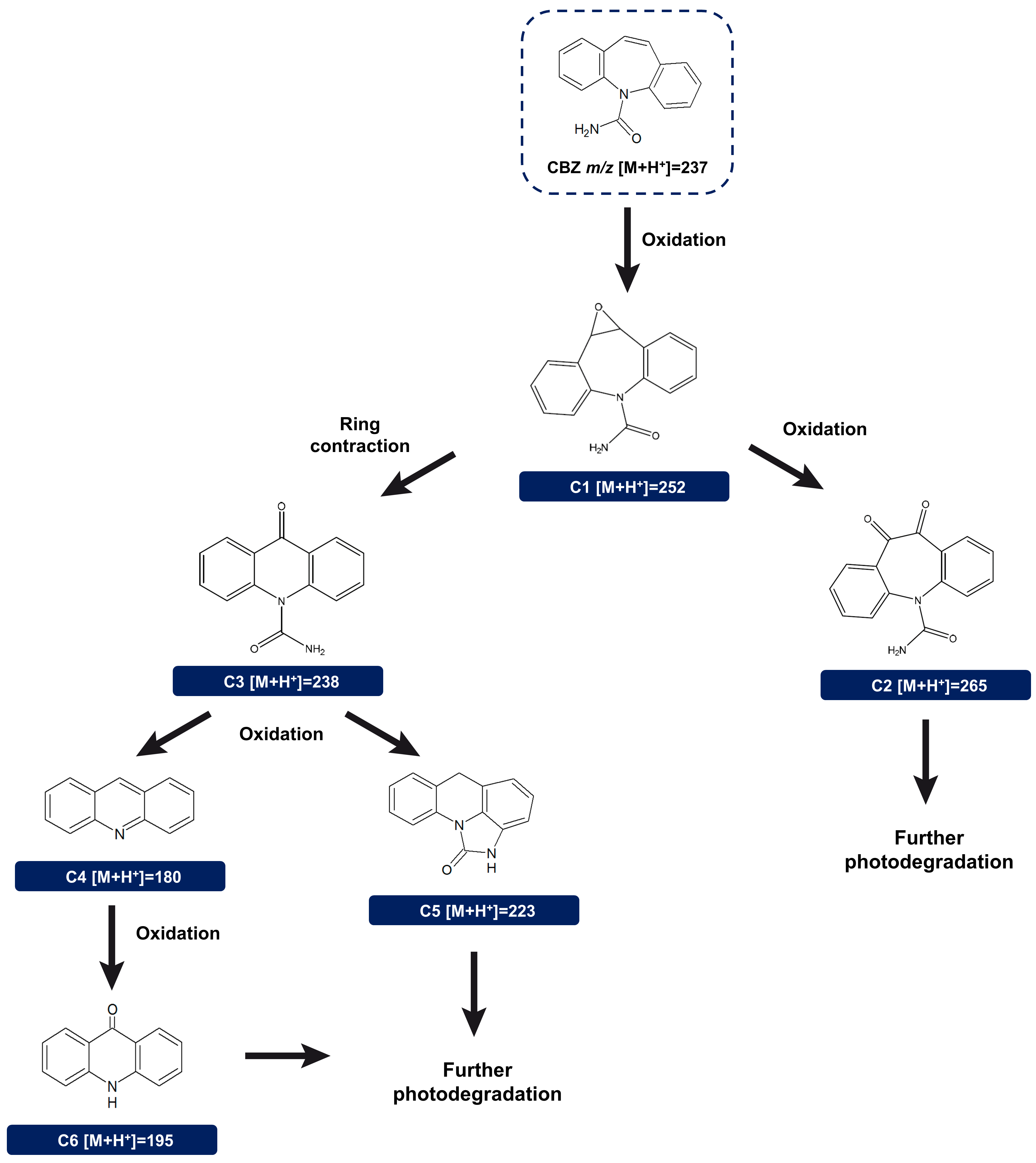


**Fig. S9.** Proposed degradation pathway of carbamazepine in the presence of TiO_2_-Y photocataly
